# Gene Expression Changes in the Spleen, Lungs, and Liver of Wistar Rats Exposed to β-Emitted ^31^SiO_2_ Particles

**DOI:** 10.3390/ijms26062693

**Published:** 2025-03-17

**Authors:** Nariaki Fujimoto, Nurislam Mukhanbetzhanov, Sanzhar Zhetkenev, Laura Chulenbayeva, Timur Fazylov, Mikhail Mukhortov, Hitoshi Sato, Kassym Zhumadilov, Valeriy Stepanenko, Andrey Kaprin, Sergey Ivanov, Peter Shegay, Masaharu Hoshi, Almagul Kushugulova

**Affiliations:** 1 Institute for Radiation Biology and Medicine, Hiroshima University, Hiroshima 734-8553, Japan; 2 Center for Life Sciences, National Laboratory Astana, Nazarbayev University, Astana Z05H0P9, Kazakhstan; nurislam.mukhanbetzhanov@nu.edu.kz (N.M.); sanzhar.zhetkenev@nu.edu.kz (S.Z.); laura.chulenbayeva@nu.edu.kz (L.C.); akushugulova@nu.edu.kz (A.K.); 3 B. Atchabarov Scientific Research Institute of Fundamental and Applied Medicine, Asfendiyarov Kazakh National Medical University, Tole Bi Street 94, Almaty 050012, Kazakhstan; timson1193@mail.ru; 4 Institute of Nuclear Physics of the Ministry of Energy of the Republic of Kazakhstan, Almaty 050032, Kazakhstan; m.mukhortov@inp.kz; 5 Faculty of Health Sciences, Ibaraki Prefectural University of Health Sciences, Ami-machi, Inashiki-gun, Ibaraki 300-0394, Japan; ipu.sato@gmail.com; 6 Department of Nuclear Physics, L.N. Gumilyov Eurasian National University, Astana 010000, Kazakhstan; zhumadilovk@gmail.com; 7 A. Tsyb Medical Radiological Research Center, Branch of the National Medical Research Radiological Centre of the Ministry of Health of the Russian Federation, 4 Koroleva St., Obninsk, Kaluga 249036, Russia; valerifs@yahoo.com (V.S.); mrrc@mrrc.obninsk.ru (S.I.); 8 National Medical Research Radiological Centre of the Ministry of Health of the Russian Federation, Koroleva Str., 4., Obninsk, Kaluga 249036, Russia; mnioi.nauka@mail.ru (A.K.); contact.shegai@mail.ru (P.S.); 9 Peoples’ Friendship University of Russia, 6 Miklukho-Maklaya St., Moscow 117198, Russia; 10 P.A. Hertzen Moscow Oncology Research Institute-Branch of the National Medical Research Radiological Centre of the Ministry of Health of the Russian Federation, 2nd Botkinsky Drive 3, Moscow 125284, Russia; 11 The Center for Peace, Hiroshima University, Higashisenda-machi 1-1-89, Naka-ku, Hiroshima 730-0053, Japan; mhoshi@hiroshima-u.ac.jp

**Keywords:** ^31^Si, ^31^SiO_2_ microparticles, internal irradiation, residual radiation, atomic bomb

## Abstract

To understand the biological effects of residual radioactivity after the atomic bomb explosion in Hiroshima and Nagasaki, we previously investigated the effects of ^56^Mn, a major residual radioisotope. Our rat study demonstrated that inhalation exposure to ^56^MnO_2_ microparticles affected gene expression in the lungs, testes, and liver, despite the low radiation doses. Because ^56^Mn is a β- and γ-emitter, the differential effects between β- and γ-rays should be clarified. In this study, ^31^Si, a β-emitter with a radioactive half-life similar to that of ^56^Mn, was used to determine its effects. Male Wistar rats were exposed to sprayed neutron-activated ^31^SiO_2_ microparticles, stable SiO_2_ microparticles, or X-rays. The animals were examined on days 3 and 14 after irradiation. The expression of radiation-inducible marker genes, including *Ccng1*, *Cdkn1a*, and *Phlda3*, was measured in the spleen, lungs, and liver. Furthermore, the expressions of pathophysiological marker genes, including *Aqp1*, *Aqp5*, and *Smad7* in the lungs and *Cth*, *Ccl2*, and *Nfkb1* in the liver, were determined. Impacts of ^31^SiO_2_ exposure were observed mainly in the liver, where the expression of *Cth* markedly increased on post-exposure days 3 and 14. Our data suggest that internal exposure to β-emitted microparticles has significant biological effects and its possible roles as residual radiation after atomic bombing.

## 1. Introduction

To evaluate the biological effects of the atomic bombings on Hiroshima and Nagasaki, the radiation doses directly from the detonated bombs were the most significant parameters. However, individuals who entered these cities after the explosion also had symptoms similar to acute radiation syndrome and a later increase in solid cancer mortality risk [1,2]. The residual radioactive dust in the area may cause these effects. Because ^56^Mn is a radioisotope produced in soil by the neutron beams from atomic explosions, we postulated that insoluble ^56^MnO_2_ is a critical source of residual radiation that victims may have inhaled and affected [3]. Our previous investigations in laboratory rodents revealed that internal exposure to ^56^MnO_2_ microparticles significantly affected the expression of genes and proteins in the examined organs even though the absorbed radiation dose in each organ was low [4].

These studies may contribute to understanding the potential health hazards to the people exposed to radioactive particles, including nuclear industry workers and the general public-enouncing nuclear accidents. In the case of the evacuees of the Chornobyl accident, the inhalation radiation doses were about 10 times higher than the external doses, although individual dose estimation is challenging to execute [5]. In the Fukushima Daiichi Nuclear accident, the atmospherically released radionuclides were estimated to go offshore and have less impact on human exposure [6]. Studies of the biological impacts of low-dose exposure to radioactive particles are required to evaluate these events.

Our theoretical analysis in the lung model, in which ^56^MnO_2_ microparticles were postulated to attach randomly to the alveolus epithelial cells, suggested that exposure to β-particles out of ^56^Mn decays produced a sharp distance-dependent absorbed dose gradient [7]. The tissues located close to ^56^Mn particles could receive intensively higher radiation doses than the average for the entire tissue. These high radiation doses in the tissue may be key to understanding our previous findings on gene/protein expression changes following low radiation doses from ^56^MnO_2_. To isolate the effects of β-emission, we used ^31^Si, an almost pure β-emitter, in this study. ^31^Si is also an isotope created in soil by neutron beams from the atomic bomb.

Since we found the significant effects of ^56^MnO_2_ exposure in the lungs and the liver, the gene expressions of these organs were examined for comparison in the present study. In addition, the spleen, whose loss in weight is a well-known marker for radiation exposure, was examined. Male Wistar rats were exposed to sprayed ^31^SiO_2_ microparticles in the air, and gene expression in the spleen, lungs, and liver was determined on post-exposure days 3 and 14. We examined the mRNA expression of p53-regulated genes, including *Cdkn1a*, *Ccng1*, and *Phlda3*, because they are useful indicators of radiation responses [8,9,10]. We further examined the expression of pathophysiologically relevant genes, such as *Aqp1*, *Aqp5*, and *Smad7* in the lungs and *Cth*, *Ccl2*, and *Nfkb1* in the liver [4,11]. Aquaporins control the water permeability of the plasma membrane [12]. It was reported that irradiation increased the *Aqp5* and reduced *Aqp1* mRNA expressions, probably protecting the radiation-induced inflammation and edema [13]. *Smad7*, a component of the TGFβ-Smad pathway, could increase in response to radiation-induced tissue injuries [14]. In the liver, irradiation increases all three marker gene expressions to protect the tissue against oxidative stress and inflammatory changes.

## 2. Results

### 2.1. Radiation Doses

The absorbed dose of inhaled irradiation from ^31^SiO_2_ in each organ was previously reported [15]. The absorbed doses in the spleen, lungs, and liver were 0.46 ± 0.2 mGy, 11 ± 4 mGy, and 0.28 ± 0.1 mGy, respectively. The external irradiation dose of X-irradiation with a linear accelerator (LINAC) was 2.0 Gy.

### 2.2. Body, Spleen, Lung, and Liver Weights

Table 1 presents body and organ weights at necropsy on days 3 or 14 after exposure (Individual data are shown in Supplementary Table S1). Body weight steadily increased during the 14 days of the study in every group. No significant changes in body weight were observed among the groups. On post-exposure day 3, spleen weight was significantly reduced in the ^31^Si group, whereas it recovered on day 14. No significant changes in lung and liver weights were observed among the groups on either day.

### 2.3. mRNA Expression of the Radiation Dose–Response Marker Genes Cdkn1a, Ccng1, and Phlda3

**Figure 1:**
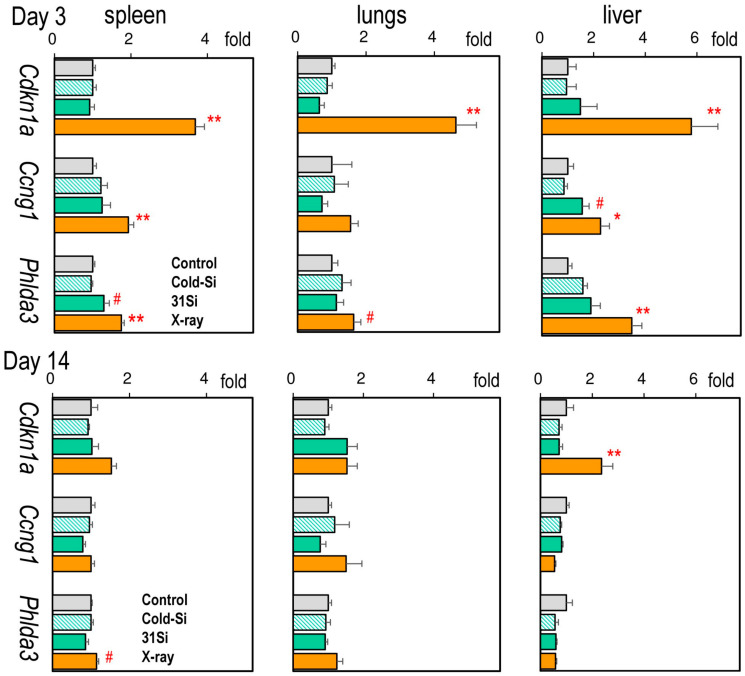
Relative mRNA levels of *Cdkn1a*, *Ccng1*, and *Phlda3* in the spleen, lungs, and liver 3 and 14 days after exposure to nonradioactive SiO_2_ particles (Cold-Si), ^31^SiO_2_ particles (31Si), or 2 Gy of X-rays (X-ray). Each bar indicates mean ± standard error of the mean (*n* = 5, each group). * *p* < 0.05 or ** *p* < 0.01 versus control. # *p* < 0.05 versus control or Cold-Si (paired comparison). X-rays increased the expression of all three marker genes on day 3, except for Ccng1 in the lung. ^31^SiO_2_ exposure increased splenic *Phlda3* and hepatic *Ccng1* expressions on day 3.

Figure 1 presents the gene expression levels of *Cdkn1a*, *Ccng1*, and *Phlda3* in the spleen, lungs, and liver (Individual data are shown in Supplementary Tables S2–S4). On post-exposure day 3, the expression of all three marker genes was induced by external X-irradiation in every organ examined, except for *Ccng1* in the lung. In contrast, only splenic *Phlda3* and hepatic *Cdkn1a* mRNA levels were significantly high on day 14. ^31^SiO_2_ exposure significantly induced the expressions of splenic *Phlda3* and hepatic *Ccng1* on day 3.

### 2.4. mRNA Expression of the Radiation-Sensitive Pathophysiological Marker Genes Aqp1, Aqp5, and Smad7 in the Lungs

**Figure 2:**
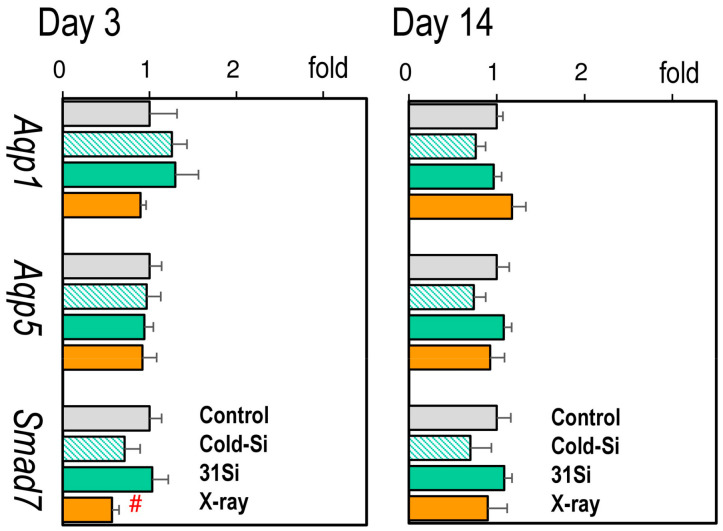
Relative mRNA levels of *Aqp1*, *Aqp5*, and *Smad7* in the lungs 3 and 14 days after exposure to nonradioactive SiO_2_ particles (Cold-Si), ^31^SiO_2_ particles (31Si), or 2 Gy of X-rays (X-ray). Each bar indicates mean ± standard error of the mean (*n* = 5, each group). # *p* < 0.05 versus control (paired comparison). X-rays reduced the expression of *Smad7*.

Figure 2 presents the expression of pulmonary pathophysiological marker genes (Individual data are accessible in Supplementary Table S3). No changes in the expression of either *Aqp1*, *Aqp5*, or *Smad7* were observed 3 or 14 days after exposure to external X-irradiation or ^31^SiO_2_, except for a reduction in the expression of *Smad7* on day 3.

### 2.5. mRNA Expression of the Radiation-Sensitive Pathophysiological Marker Genes Cth, Ccl2, and Nfkb1 in the Liver

**Figure 3:**
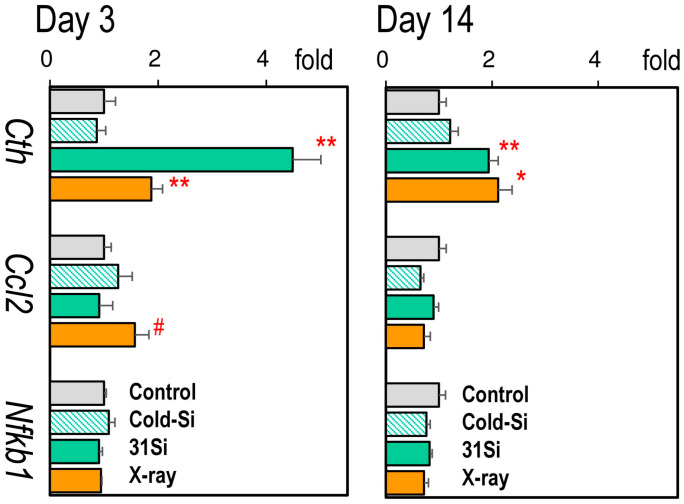
Relative mRNA levels of *Cth*, *Ccl2*, and *Nfkb1* in the liver 3 and 14 days after exposure to nonradioactive SiO_2_ particles (Cold-Si), ^31^SiO_2_ particles (31Si), or 2 Gy of X-rays (X-ray). Each bar indicates mean ± standard error of the mean (*n* = 5, each group). * *p* < 0.05 or ** *p* < 0.01 versus control. # *p* < 0.05 versus control (paired comparison). The expression of *Cth* was increased in the ^31^Si and X-ray groups on post-exposure days 3 and 14. The expression of *Ccl2* was increased in the X-ray group on day 3.

Figure 3 presents the expression of hepatic pathophysiological marker genes (Individual data are accessible in Supplementary Table S4). On post-exposure day 3, the mRNA levels of Cth were significantly increased by both external X-irradiation and ^31^SiO_2_ exposure, which interestingly continued to be high on day 14. The expression of *Ccl2* was increased on day 3 in the X-ray group.

## 3. Discussion

This study investigated the effects of internal exposure to β-ray emitting ^31^SiO_2_ microparticles on the expression of marker genes in the spleen, lungs, and liver of rats. Exposure to ^31^SiO_2_ prominently increased the mRNA expression of *Cth*, even at low radiation doses in the liver. The results demonstrated the biological significance of exposure to β-emitting particles.

To fully understand the biological effects of atomic bombing, the residual radioactivity produced by the neutron beams from explosions should be considered [16]. Our previous studies involving rodents have reported that internal exposure to ^56^MnO_2_, a residual radiation source, significantly affected the expression of genes in organs [4,17]. Although ^56^Mn emits both β- and γ-rays, our theoretical study suggested the significance of β-rays, which are associated with high local radiation doses in the small tissue areas close to each radioactive particle and may result in biological consequences despite the low whole-organ doses [7]. In this study, ^31^SiO_2_ powders were used instead of ^56^MnO_2_ to examine the biological effects of internal β-rays. ^31^Si is also a residual radioisotope produced by the neutron beams of atomic bombs and primarily emits β-rays with a radioactive half-life of approximately 2.62 h [3].

This study focused on the spleen, lungs, and liver. Spleen weights are a radiation-dose-dependent index for whole-body irradiation [18]. In the X-ray group, relative spleen weights decreased on post-exposure day 3 and returned to the control level on day 14. A similar change was observed in the ^31^Si group, although the splenic radiation dose was only 0.46 mGy. Radiation-induced spleen weight loss is associated with lymphocyte apoptosis, not only in the spleen but also in the whole body [19,20]. Spleen weight loss might result from the systemic effects of ^31^Si exposure. However, no reduction in spleen weight was observed in our previous ^56^MnO_2_ study [4]. The discrepancy needs to be addressed in future studies.

Changes in the expression of p53-regulated genes could be used as radiation exposure signatures [21,22]. A microarray analysis identified highly sensitive expression marker genes, including *Cdkn1a*, *Cccng1*, and *Phlda3* [10]. Although these markers were mainly found in studies of peripheral blood analysis, Some of them are also useful as exposure markers in solid tissues [23,24]. We then determined the expression of these genes to evaluate their responses to radiation exposure. As expected, external X-irradiation induced the expression of these marker genes on post-exposure day 3. *Cdkn1a* mRNA levels remained high on day 14, consistent with a previous finding involving mouse livers [25] and our previous study [17]. ^31^Si exposure increased the expression of *Ccng1* in the liver, indicating that the liver responded to radiation from ^31^Si, although the gene was not radiation-inducible in the lung.

The lungs are the primary targets of ^31^Si because animals inhale sprayed ^31^SiO_2_ particles in the air. To examine the possible biological consequences of ^31^Si, three pathophysiological marker genes were examined: *Aqp1*, *Aqp5*, and *Smad7*. *Aqp1* and *Aqp5* encode water-selective channel proteins, aquaporins, that play critical roles in maintaining lung structure and function [12]. The altered expression in *Aqp1* and *Aqp5* represents physiological and pathological changes in the lungs exposed to radiation or infection [13,26]. The expression of *Smad7* may increase during the healing process from radiation-induced tissue damage [14]. Our previous study demonstrated that exposure to ^56^MnO_2_ increased the expression of *Aqp5* and *Smad7* in the lungs, whereas 2 Gy of external irradiation did not [4]. However, in this study, no such changes were found in the lungs in the ^31^Si group, probably because of the differences in the lung doses between the two studies (48–68 mGy in the ^56^Mn study vs. 10 mGy in the present ^31^Si experiment).

Radiation exposure to the liver can cause hepatic injuries. Irradiation at high radiation doses (>30 Gy) results in the development of liver fibrosis, whereas lower doses affect hepatic function by inducing inflammatory and oxidative stresses, in which the expression of cytokines, such as IL-6 and CCL2, increases with the activation of the NFκb signaling [27,28]. Thus, these gene expression changes may serve as useful markers for evaluating radiation-induced hepatic damage; however, no indications were found in the ^31^Si group. An investigation of human hepatic cultured cells revealed that the expression of *Cth* was inducible by irradiation [11]. A protein encoded by *Cth* is an enzyme, cystathionine γ-lyase, producing hydrogen sulfide that protects hepatocytes against oxidative stress. This ability renders *Cth* an excellent expression marker for radiation-induced hepatic injuries. This study demonstrated that X-irradiation induced the expression of *Cth* in the liver not only in the cultured cells but also in vivo. The expression of *Cth* remained high on post-exposure day 14, which made it a more stable marker representing hepatic responses to radiation. Interestingly, ^31^SiO_2_ exposure also increased the expression of *Cth* in the liver, which was higher than that in the X-ray group, suggesting that ^31^SiO_2_ microparticles have more intensive biological effects on the liver. The exact physiological functions of *Cth* in the hepatocytes need to be defined in the future.

Our dosimetry results revealed a very low hepatic radiation dose averaged over whole-organ volume (0.28 mGy), calculated from the radioactivity measured 30 min after exposure. Most inhaled ^31^SiO_2_ particles rapidly moved from the bronchus to the digestive tract as the large intestinal dose reached 120 mGy [15]. An oral kinetics study showed that the plasma level of SiO_2_ started to increase 90 min after oral administration, suggesting that the hepatic dose of ^31^SiO_2_ through the portal vein system could elevate later than our timing of the radioactivity measurement, even considering the short radioactive half-life of ^31^Si. Furthermore, insoluble microparticles could be taken up across the mucosal barrier of the intestinal tract and transported to other organs, particularly the liver, where they are excreted or further distributed [29,30]. Thus, ^31^SiO_2_ microparticles can enter the liver and cause significant adverse effects on hepatocytes located close to the microparticles, as predicted by our previous theoretical analysis [8]. A study of 90Y microsphere treatment showed that administered microparticles were localized in the hepatic artery and trapped within the terminal arterioles [31]. A recent investigation of orally exposed polymer microparticles demonstrates their uniform distribution in the liver tissue [32]. Further studies are necessary to examine the details of ^31^SiO^2^ particle transport from the gastrointestinal tract and the distribution in the liver tissue and to estimate the peculiarities of local internal doses on the level of the liver’s microstructures.

The chemical toxicity of SiO_2_ has been extensively studied [33]. Its oral toxicity is considered to be very low because this compound exists ubiquitously in the environment. However, the health hazards of inhaled crystalline SiO_2_ are well documented, including silicosis and other pulmonary diseases [34,35]. The amorphous SiO_2_, used in this study possesses little toxicity, although chronic inhalation of amorphous SiO_2_ microparticles or nanoparticles may increase the expression of proinflammatory markers in the alveoli [35]. No reports have suggested that chemical toxicity could arise from the 1 h inhalation of SiO_2_ microparticles.

A recent study showed that ^137^Cs-bearing particles could cause DNA damage in the cells closely located to the particles using the cell culture [36]. A similar in vitro setup combined with a single-cell gene expression analysis will help examine our hypothesis of distant-dependent gene expression changes in the cells closely located to β-emitting particles in the future. It is unclear if our findings in gene expression changes have any specific implications for human health. The future global gene expression analysis in the ^31^Si exposed animals could provide a more complete view of the effects and possible relevance to human health.

## 4. Materials and Methods

### 4.1. Animals

The animal study was approved by the Animal Experiment Ethics Committee of Asfendiyarov Kazakh National Medical University, Almaty, Kazakhstan (document #1630, 7 September 2023). The study was conducted in accordance with the Animal Research: Reporting of In Vivo Experiments (ARRIVE) guidelines. Ten-week-old male Wistar rats were obtained from Asfendiyarov Kazakh National Medical University, Almaty, Kazakhstan. The animals were maintained in cages with free access to a basal diet and tap water. After a week of acclimatation, the rats were randomly divided into four groups: the control group (n = 10), Cold-Si group (n = 13), ^31^Si group (n = 13), and X-ray group (n = 10), with a total n = 46. The Cold-Si group was exposed to stable SiO_2_ microparticles. The ^31^Si group was exposed to ^31^SiO_2_ microparticles at an activity of 3.2 × 10^7^ Bq/g. Three rats from the ^31^Si group were used to estimate the absorbed radiation doses. The X-ray group received 2 Gy of external X-rays using a LINAC. Five rats from each group underwent necropsies on post-exposure days 3 and 14. The rats were euthanized by removal of whole blood under anesthesia with isoflurane (Fujifilm Wako Pure Chemical Co., Tokyo, Japan). The spleen, lungs, and liver were dissected, and tissue pieces were stored in Gene Keeper solution (Fujifilm Wako Pure Chemical Co.) for RNA and protein extraction.

### 4.2. Irradiation and Dosimetry

Exposure to ^31^SiO_2_ microparticles and internal dose estimation of internal doses averaged over whole organs’ volumes were performed as previously described [15]. In brief, SiO_2_ powder (Rare Metallic Co., Tokyo, Japan; average diameter of the particles: 2.4 µm, purity: >99.9%) was activated by a neutron beam in the WWR-K nuclear reactor at the Institute of Nuclear Physics, Almaty, Kazakhstan. The obtained radioactive ^31^SiO_2_ powder was sprayed over the rats for 1 h. Then, three rats of the ^31^Si group were euthanized for β-spectrometry, and each organ’s absorbed dose was estimated. All animals were brought to the exposure facility to maintain the same environment in each group. A medical LINAC, Clinac 2100C/D (Varian Medical Systems Inc., Palo Alto, CA, USA), was used for whole-body γ-irradiation at a dose rate of 6 Gy/min.

### 4.3. QRT Polymerase Chain Reaction (PCR)

Total RNA was isolated from tissues stored in Gene Keep solution with Isogen II (Nippon Gene Co., Tokyo, Japan). cDNA was obtained by incubating 3 µg of total RNA + RevaTra Ace 100 U (Toyobo Co., Osaka, Japan) + 20 pmol random hexamers/5 pmol oligo-dT(15) primers (Takara Bio Inc., Kusatsu, Japan). The quantitative PCR system StepOnePlus (Applied Biosystems, Life Technologies, Co., Carlsbad, CA, USA) was used for cDNA measurement with Thunderbird Next Sybr q-PCR Mix (Toyobo Co.). PCR was performed with a 2-min initial denaturation, followed by 40 cycles of 5 s at 95 °C and 35 s at 60 °C. Table 2 presents the specific primer sets for the genes. The nucleotide sequence of each PCR product was verified by Fasmac Co., Ltd. (Atsugi, Japan). The mRNA levels were normalized with reference to the β-actin mRNA levels.

### 4.4. Statistical Analysis

For multiple comparisons among the groups, Dunnett’s test was used with R’s package “SimComp” version 3.3 (http://cran.r-project.org, accessed on 10 December 2024). Paired comparisons were performed with the *t*-test function of the Microsoft Excel 2019 program.

## 5. Conclusions

This study investigated the effects of exposure to ^31^SiO_2_ microparticles emitting predominantly β-rays on the gene expression in the spleen, lungs, and liver of Wistar rats on post-exposure days 3 and 14. ^31^SiO_2_ exposure induced little expression change in the spleen or lungs. However, it markedly increased the expression of *Cth* in the liver on postexposure days 3 and 14, even at very low values of internal radiation doses averaged over whole organs’ volume. This indicates the significant biological effects of β-emitting particles and their possible roles as residual radiation of atomic bombs. In order to better understanding these effects, the estimations of local values of internal dose from exposure to ^31^SiO_2_ microparticles on the liver’s microstructure are ongoing.

## Figures and Tables

**Table 1:** Body and the relative organ weights in rats exposed to cold SiO_2_, ^31^SiO_2_, or γ-rays.

Groups		Initial Body Weight (g)	Body Weight (g)	Spleen (g/kg bw)	Lung (g/kg bw)	Liver (g/kg bw)
Day 3	Control	271 ± 30	277 ± 29	4.8 ± 0.84	8.4 ± 0.46	28 ± 1.9
	Cold-Si	298 ± 27	297 ± 22	4.2 ± 0.30	9.9 ± 2.47	31 ± 1.7
	31Si	303 ± 26	309 ± 26	3.2 ± 0.36 ^#^	8.4 ± 1.38	29 ± 1.1
	X-ray	283 ± 27	288 ± 26	3.6 ± 0.4	11.6 ± 4.17	29 ± 1.1
Day 14	Control	293 ± 33	314 ± 29	3.5 ± 0.22	8.5 ± 1.05	26 ± 0.7
	Cold-Si	291 ± 39	300 ± 39	4.1 ± 0.60	10.2 ± 0.85	30 ± 1.0
	31Si	299 ± 37	319 ± 37	4.3 ± 0.64	9.2 ± 0.75	31 ± 1.9
	X-ray	295 ± 34	319 ± 38	3.3 ± 0.25	12.9 ± 3.84	32 ± 1.7

Control: untreated group; Cold-Si: exposed to stable SiO_2_ particles; 31Si: exposed to ^31^SiO^2^ particles at the activity of 4.9 × 10^7^ Bq/g; X-ray: exposed to X-rays (2 Gy) with an LINAC. Initial body weights are the body weights a day before exposure. Each value shows mean ± standard error of the mean (*n* = 5, each group). ^#^ indicates a significant difference from the Cold-Si at *p* < 0.05 (paired comparison).

**Table 2:** Q-PCR primers.

Gene	GenBank Accession #	Q-PCR Primer Sequences (5′ -> 3′)	
		Forward	Reverse
*Cdkn1a*	NM_080782	TGTCCGACCTGTTCCACACA	CGTCTCAGTGGCGAAGTCAA
*Ccng1*	NM_012923	CGTGCCACTGCAGGATCATA	AAGGTCAGATCTCGGCCACTT
*Phlda3*	NM_001012206	AAGCCGTGGAGTGCGTGGAGAG	GTCTGGATGGCCTGTTGATTC
*Aqp1*	NM_012778	CCACTGGAGAGAAACCAGACG	CTGAGCAGAAGCCCCAGTGT
*Aqp5*	NM_012779	ATGCGCTGAACAACAACACAAC	GTGACAGACAAGCCAATGGATAAG
*Smad7*	NM_030858	TTGCTGTGAATCTTACGGGAAG	GGTTTGAGAAAATCCATCGGGT
*Cth*	NM_017074	TCCCCGGGTAGAAAAGGTTATT	TTGAGGAAGACCTGAGCATGC
*Ccl2*	NM_031530	AAGCCAGATCTCTCTTCCTCCA	CAGCAACTGTGAACAACAGGC
*Nfkb*	NM_001276711	GGGCTACACAGAGGCCATTG	TCTCGGAGCTCATCTATGTGCT
*Actb*	NM_031144.3	TTGTCCCTGTATGCCTCTGGTC	TGAGGTAGTCTGTCAGGTCCC

## Data Availability

All data are presented in the article and the Supplementary Materials.

## Supplementary Materials

The following supporting information can be downloaded at: https://www.mdpi.com/article/10.3390/ijms26062693/s1.
