# Supplementary material for: Gene Expression Changes in the Spleen, Lungs, and Liver of Wistar Rats Exposed to β-Emitted 31SiO2 Particles
Source: Int J Mol Sci. 2025 Mar 17;26(6):2693. doi: 10.3390/ijms26062693 (PMC11942150; doi:10.3390/ijms26062693)
Supplement: Supplementary file 1 [file ijms-26-02693-s001.zip › TableS4.pdf]

Table S4 - QRT-PCR data of the liver of rats exposed to 31SiO2

Liver

		bactin	Cdkn1a			Ccng1			Phlda3		
Day3		(fg)	(fg)	x100/bactin	fold change	(fg)	x10/bactin	fold change	(fg)	x100/bactin	fold change
Control	#41	17.8	0.43	2.43	0.48	27.13	15.27	0.82	1.14	6.41	1.00
	#42	7.7	0.16	2.02	0.40	12.24	15.90	0.85	0.41	5.31	0.83
	#43	7.6	0.25	3.30	0.66	5.37	7.05	0.38	0.24	3.19	0.49
	#44	12.2	1.29	10.60	2.10	28.83	23.62	1.26	1.11	9.06	1.41
	#45	6.2	0.42	6.85	1.36	19.58	31.65	1.69	0.51	8.22	1.28
			Cnrl Avg=>	5.04	1.00	Cnrl Avg=>	18.69	1.00	Cnrl Avg=>	6.44	1.00
				SEM	0.32			0.22			0.16
Cold-Si	#21	11.0	0.42	3.81	0.76	18.82	17.06	0.91	1.18	10.72	1.67
	#22	12.9	0.29	2.27	0.45	20.29	15.78	0.84	1.00	7.80	1.21
	#23	4.5	0.11	2.46	0.49	5.07	11.34	0.61	0.47	10.44	1.62
	#24	5.1	0.16	3.20	0.63	5.60	10.92	0.58	0.42	8.13	1.26
	#25	7.0	0.86	12.27	2.43	17.37	24.65	1.32	0.98	13.96	2.17
				Mean	0.95			0.85			1.59
				SEM	0.37			0.13			0.17
				Dunnett p	0.854			0.909			0.058
31Si	#31	8.7	0.22	2.53	0.50	14.23	16.27	0.87	0.50	5.71	0.89
	#32	4.9	0.17	3.49	0.69	13.93	28.16	1.51	0.58	11.72	1.82
	#33	5.6	0.45	8.04	1.59	18.93	33.58	1.80	0.87	15.39	2.39
	#34	12.5	0.43	3.47	0.69	28.50	22.81	1.22	1.13	9.05	1.41
	#35	12.8	2.56	19.97	3.96	57.83	45.19	2.42	2.45	19.12	2.97
				Mean	1.49			1.56			1.90
				SEM	0.65			0.26			0.36
				Dunnett p	0.548			0.162			0.095
				vs Cold-Si p	0.247			0.021			0.233
X-ray	#11	10.6	2.00	18.88	3.75	24.11	22.75	1.22	1.39	13.08	2.03
	#12	11.7	2.53	21.68	4.30	49.89	42.68	2.28	2.75	23.53	3.66
	#13	11.1	3.59	32.34	6.42	43.79	39.48	2.11	2.70	24.33	3.78
	#14	14.5	3.62	24.94	4.95	63.77	43.96	2.35	3.30	22.72	3.53
	#15	13.6	6.50	47.89	9.50	85.68	63.17	3.38	3.84	28.28	4.39
				Mean	5.78			2.27			3.48
				SEM	1.03			0.34			0.39
				Dunnett p	0.015			0.026			0.003
				vs Ctrl p	0.001			0.007			0.000
Day14		(fg)	(fg)	x100/bactin	fold change	(fg)	x10/bactin	fold change	(fg)	x100/bactin	fold change
Control	#46	6.6	0.19	2.89	0.52	9.75	14.71	0.60	0.39	5.85	0.44
	#47	8.8	0.35	4.02	0.72	22.98	26.05	1.06	1.81	20.54	1.56
	#48	10.8	0.62	5.77	1.03	26.35	24.37	0.99	0.65	6.00	0.45
	#49	9.4	0.36	3.79	0.68	27.36	29.22	1.19	1.49	15.95	1.21
	#40	5.5	0.64	11.60	2.07	15.55	28.28	1.15	0.97	17.59	1.33
			Cnrl Avg=>	5.62	1.00	Cnrl Avg=>	24.52	1.00	Cnrl Avg=>	13.18	1.00
					0.28			0.11			0.23
Cold-Si	#26	15.3	0.76	5.00	0.89	30.23	19.80	0.81	1.32	8.66	0.66
	#27	15.2	0.41	2.66	0.47	24.89	16.33	0.67	0.75	4.93	0.37
	#28	17.5	0.41	2.34	0.42	36.22	20.64	0.84	0.78	4.45	0.34
	#29	21.5	0.96	4.46	0.79	31.20	14.53	0.59	1.19	5.52	0.42
	#30	14.6	0.83	5.70	1.02	32.43	22.19	0.90	1.99	13.61	1.03
				Mean	0.72			0.76			0.56
				SEM	0.12			0.06			0.13
				Dunnet p	0.933			0.981			0.956
31Si	#36	12.4	0.61	4.97	0.88	30.39	24.58	1.00	0.87	7.00	0.53
	#37	19.4	0.62	3.20	0.57	33.38	17.23	0.70	1.27	6.58	0.50
	#38	14.7	0.51	3.46	0.62	25.71	17.45	0.71	1.20	8.13	0.62
	#39	13.1	0.84	6.45	1.15	27.23	20.81	0.85	1.28	9.80	0.74
	#40	20.6	0.44	2.13	0.38	41.66	20.22	0.82	1.60	7.78	0.59
				Mean	0.72			0.82			0.60
				SEM	0.13			0.05			0.04
				Dunnett p	0.930			0.962			0.952
				vs Cold-Si p	0.497			0.252			0.409
X-ray	#16	22.5	1.55	6.88	1.22	27.69	12.28	0.50	1.93	8.55	0.65
	#17	14.1	1.98	14.00	2.49	17.53	12.40	0.51	0.90	6.37	0.48
	#18	11.5	2.41	20.95	3.73	17.15	14.89	0.61	1.01	8.79	0.67
	#19	7.7	0.72	9.33	1.66	7.93	10.30	0.42	0.47	6.04	0.46
	#20	4.7	0.71	15.12	2.69	8.03	17.04	0.69	0.41	8.65	0.66
				Mean	2.36			0.55			0.58
				SEM	0.43			0.05			0.05
				Dunnett p	0.042			0.998			0.954
				vs Ctrl p	0.015			0.002			0.058

Liver

Cth (fg)	x100/bactin	fold change	Ccl2 (fg)	x100/bactin	fold change	Nfkb1 (fg)	x100/bactin	fold change
6.46	36.37	0.97	1.16	6.55	1.21	0.20	1.11	1.00
2.64	34.23	0.91	0.43	5.54	1.02	0.08	1.10	0.98
1.07	13.98	0.37	0.56	7.29	1.35	0.10	1.28	1.15
4.88	39.98	1.06	0.53	4.31	0.79	0.12	1.01	0.91
3.94	63.64	1.69	0.21	3.41	0.63	0.07	1.07	0.96
Cnrl Avg=>	37.64	1.00	Cnrl Avg=>	5.42	1.00	Cnrl Avg=>	1.11	1.00
		0.21			0.13			0.04
5.62	50.92	1.35	0.48	4.36	0.80	0.11	0.97	0.87
5.44	42.29	1.12	0.68	5.32	0.98	0.12	0.92	0.83
1.18	26.48	0.70	0.44	9.76	1.80	0.07	1.51	1.36
0.80	15.61	0.41	0.55	10.65	1.96	0.08	1.49	1.34
1.94	27.46	0.73	0.29	4.12	0.76	0.08	1.16	1.04
	Mean	0.86			1.26			1.09
	SEM	0.17			0.26			0.11
	Dunnet p	0.914			0.445			0.492
13.10	149.75	3.98	0.41	4.64	0.86	0.09	1.03	0.93
10.62	214.69	5.70	0.16	3.32	0.61	0.05	0.95	0.85
6.32	112.18	2.98	0.57	10.08	1.86	0.07	1.27	1.14
19.56	156.56	4.16	0.54	4.36	0.80	0.11	0.87	0.78
27.06	211.42	5.62	0.30	2.31	0.43	0.12	0.93	0.83
	Mean	4.49			0.91			0.91
	SEM	0.52			0.25			0.06
	Dunnett p	0.002			0.905			0.984
	vs Cold-Si p	0.000			0.178			
6.56	61.88	1.64	0.83	7.84	1.45	0.12	1.09	0.98
6.81	58.24	1.55	0.90	7.70	1.42	0.13	1.07	0.96
7.22	65.13	1.73	1.55	14.00	2.58	0.12	1.06	0.95
9.63	66.36	1.76	0.97	6.69	1.23	0.14	0.97	0.87
13.72	101.16	2.69	0.86	6.34	1.17	0.14	1.06	0.95
	Mean	1.87			1.57			0.94
	SEM	0.21			0.26			0.02
	Dunnet p	0.027			0.133			0.984
	vs Ctrl p	0.009			0.042			0.116

(fg)	x100/bactin	fold change	(fg)	x100/bactin	fold change	(fg)	x100/bactin	fold change
2.39	36.04	0.85	0.55	8.27	1.09	0.09	1.33	1.04
5.24	59.39	1.39	0.96	10.91	1.44	0.15	1.68	1.31
2.73	25.22	0.59	0.60	5.59	0.74	0.09	0.79	0.61
4.55	48.57	1.14	0.49	5.27	0.70	0.10	1.09	0.85
2.42	43.99	1.03	0.43	7.90	1.04	0.08	1.52	1.19
Cnrl Avg=>	42.64	1.00	Cnrl Avg=>	7.59	1.00	Cnrl Avg=>	1.28	1.00
		0.14			0.13			0.12
6.62	43.36	1.02	0.93	6.08	0.80	0.15	0.97	0.76
5.85	38.36	0.90	0.82	5.39	0.71	0.11	0.73	0.57
9.23	52.59	1.23	0.90	5.13	0.68	0.23	1.29	1.01
10.39	48.41	1.14	0.70	3.24	0.43	0.20	0.91	0.71
11.03	75.51	1.77	0.71	4.86	0.64	0.15	0.99	0.77
	Mean	1.21			0.65			0.76
	SEM	0.15			0.06			0.07
	Dunnet p	0.354			0.992			0.971
12.34	99.80	2.34	0.82	6.66	0.88	0.13	1.07	0.84
11.69	60.35	1.42	1.54	7.96	1.05	0.20	1.04	0.81
14.06	95.47	2.24	0.71	4.85	0.64	0.15	1.05	0.82
11.53	88.13	2.07	0.75	5.74	0.76	0.16	1.25	0.98
14.31	69.48	1.63	1.82	8.86	1.17	0.18	0.86	0.67
	Mean	1.94			0.90			0.82
	SEM	0.18			0.10			0.05
	Dunnett p	0.005			0.874			0.947
	vs Cold-Si p	0.007			0.062			0.256
22.02	97.67	2.29	1.27	5.64	0.74	0.14	0.60	0.47
8.66	61.28	1.44	0.69	4.88	0.64	0.13	0.89	0.69
13.75	119.41	2.80	0.48	4.14	0.55	0.12	1.05	0.82
5.22	67.81	1.59	0.29	3.71	0.49	0.06	0.83	0.65
4.95	105.06	2.46	0.41	8.80	1.16	0.06	1.24	0.96
	Mean	2.12			0.72			0.72
	SEM	0.26			0.12			0.08
	Dunnett p	0.011			0.978			0.981
	vs Ctrl p	0.003			0.129			0.161
