# Supplementary material for: Gene Expression Changes in the Spleen, Lungs, and Liver of Wistar Rats Exposed to β-Emitted 31SiO2 Particles
Source: Int J Mol Sci. 2025 Mar 17;26(6):2693. doi: 10.3390/ijms26062693 (PMC11942150; doi:10.3390/ijms26062693)
Supplement: Supplementary file 1 [file ijms-26-02693-s001.zip › TableS1.pdf]

Table S1 - Body and organ weights of rats exposed to 31SiO2

		weight (g)					relative weight (g/kg)		
Day3	Animal#	Body (day-1)	Body	Spleen	lung	Liver	Spleen	lung	Liver
Control	#41	201	211	1.67	1.92	6.73	7.91	9.08	31.91
	#42	265	292	1.46	2.07	9.07	4.99	7.09	31.07
	#43	212	218	0.98	2.12	6.41	4.50	9.72	29.42
	#44	318	297	1.11	2.41	7.88	3.73	8.10	26.53
	#45	360	366	1.10	2.88	7.84	3.00	7.88	21.42
	Mean	271	277				4.82	8.37	28.07
	SEM	30	29				0.84	0.46	1.90
Cold-Si	#21	245	232	0.88	4.55	7.18	3.79	19.57	30.88
	#22	260	285	1.15	2.30	9.31	4.04	8.07	32.67
	#23	277	295	1.49	2.70	9.74	5.05	9.15	33.02
	#24	312	303	1.44	2.00	7.36	4.75	6.60	24.29
	#25	397	371	1.28	2.31	12.19	3.45	6.23	32.86
	Mean	298	297				4.21	9.92	30.74
	SEM	27	22				0.30	2.47	1.66
	Dunnett p	0.9051	0.8851				0.38	0.97	0.94
31Si	#31	270	326	1.07	1.69	9.52	3.28	5.20	29.21
	#32	260	269	1.09	3.46	6.99	4.07	12.85	25.99
	#33	277	256	1.01	2.35	8.35	3.93	9.16	32.60
	#34	308	293	0.80	2.61	8.78	2.72	8.91	29.95
	#35	401	400	0.86	2.28	11.51	2.16	5.70	28.78
	Mean	303	309				3.23	8.36	29.30
	SEM	26	26				0.36	1.38	1.06
	Dunnett p	0.9263	0.9287				0.12	0.86	0.86
	vs Cold-Si p	0.45	0.37				0.03	0.30	0.24
X-rays	#11	203	221	0.87	1.61	6.28	3.91	7.28	28.39
	#12	260	267	0.96	7.42	8.61	3.58	27.79	32.26
	#13	275	261	0.85	1.52	7.66	3.24	5.83	29.34
	#14	314	321	1.62	3.63	9.38	5.05	11.29	29.22
	#15	365	369	0.85	2.14	9.42	2.30	5.79	25.54
	Mean	283	288				3.62	11.60	28.95
	SEM	27	26				0.45	4.17	1.08
	Dunnett p	0.8257	0.8195				0.20	0.98	0.82
	vs Ctrl p	0.39	0.39				0.12	0.23	0.35
Day 14	Animal#		Body W	Spleen	lung	Liver			
Control	#46	216	238	0.73	1.95	6.42	3.08	8.20	26.98
	#47	256	307	1.15	3.81	8.28	3.73	12.40	26.96
	#48	279	328	1.34	2.46	8.13	4.09	7.51	24.78
	#49	303	283	0.84	2.41	7.75	2.98	8.50	27.37
	#50	410	412	1.55	2.52	9.88	3.76	6.13	23.98
	Mean	293	314				3.53	8.55	26.02
	SEM	33	29				0.22	1.05	0.68
Cold-Si	#26	231	264	1.15	3.07	8.04	4.36	11.61	30.46
	#27	208	245	1.56	2.21	7.94	6.35	9.03	32.39
	#28	287	222	0.84	2.82	7.09	3.77	12.71	31.94
	#29	297	328	0.99	2.72	9.07	3.03	8.30	27.64
	#30	431	441	1.38	4.03	12.23	3.14	9.15	27.72
	Mean	291	300				4.13	10.16	30.03
	SEM	39	39				0.60	0.85	1.01
	Dunnett p	0.7518	0.6801				0.98	0.98	1.00
31Si	#36	233	311	0.94	3.52	9.87	3.02	11.33	31.73
	#37	240	257	0.88	2.25	7.35	3.43	8.77	28.61
	#38	287	317	1.37	3.12	9.20	4.33	9.85	29.02
	#39	295	254	1.00	2.36	9.68	3.93	9.29	38.11
	#40	438	457	3.06	3.07	12.49	6.69	6.72	27.32
	Mean	299	319				4.28	9.19	30.96
	SEM	37	37				0.64	0.75	1.93
	Dunnett p	0.8043	0.8239				0.98	0.91	1.00
	vs Cold-Si p	0.44	0.37				0.43	0.21	0.34
X-ray	#16	220	251	0.89	2.34	9.09	3.55	9.32	36.23
	#17	249	271	1.15	3.58	8.84	4.25	13.19	32.63
	#18	286	359	1.05	2.61	10.59	2.92	7.28	29.50
	#19	299	265	0.80	7.32	9.39	3.03	27.62	35.44
	#20	420	449	1.33	3.18	12.16	2.97	7.08	27.08
	Mean	295	319				3.34	12.90	32.17
	SEM	34	38				0.25	3.84	1.74
	Dunnett p	0.7801	0.8221				0.61	0.97	1.00
	vs Ctrl p	0.48	0.46				0.30	0.15	0.01
