# Supplementary material for: Gene Expression Changes in the Spleen, Lungs, and Liver of Wistar Rats Exposed to β-Emitted 31SiO2 Particles
Source: Int J Mol Sci. 2025 Mar 17;26(6):2693. doi: 10.3390/ijms26062693 (PMC11942150; doi:10.3390/ijms26062693)
Supplement: Supplementary file 1 [file ijms-26-02693-s001.zip › TableS3.pdf]

Table S3 - QRT-PCR data of the lungs of rats exposed to 31SiO2

Lungs

		bactin	Cdkn1a			Ccng1			Phlda3		
Day3		(fg)	(fg)	x1000/bact	fold change	(fg)	x10/bact	fold change	(fg)	x1000/bact	fold change
Control	#41	82.7	2.16	26.1	1.24	66.1	8.00	0.43	2.07	25.0	1.04
	#42	87.3	1.63	18.6	0.89	82.1	9.40	0.50	1.40	16.0	0.66
	#43	50.2	0.97	19.4	0.92	36.1	7.18	0.39	1.20	23.9	0.99
	#44	10.4	0.26	25.1	1.19	64.7	62.25	3.34	0.41	39.3	1.63
	#45	41.8	0.66	15.9	0.76	26.6	6.37	0.34	0.69	16.4	0.68
			Cnrl Avg=>	21.02	1.00	Cnrl Avg=>	18.64	1.00	Cnrl Avg=>	24.13	1.00
				SEM	0.09			0.59			0.18
Cold Si	#21	4.0	0.07	18.1	0.86	29.9		0.00	0.22	55.4	2.30
	#22	64.4	1.93	29.9	1.42	82.8	12.86	0.69	1.17	18.1	0.75
	#23	26.6	0.42	15.9	0.76	49.6	18.69	1.00	0.81	30.4	1.26
	#24	51.8	0.77	14.9	0.71	113.7	21.96	1.18	1.31	25.2	1.05
	#25	4.0	0.05	11.9	0.56	18.6	46.40	2.49	0.11	27.5	1.14
				Mean	0.86			1.07			1.30
				SEM	0.15			0.41			0.26
				Dunnett p	0.974			0.605			0.392
31Si	#31	25.4	0.50	19.5	0.93	60.9	23.96	1.29	0.88	34.6	1.44
	#32	37.2	0.69	18.6	0.88	18.2	4.90	0.26	1.53	41.2	1.71
	#33	8.4	0.07	8.6	0.41	11.9	14.19	0.76	0.15	18.0	0.74
	#34	7.9	0.03	4.1	0.20	8.9	11.26	0.60	0.11	13.5	0.56
	#35	22.2	0.35	15.8	0.75	27.0	12.15	0.65	0.65	29.3	1.21
				Mean	0.63			0.71			1.13
				SEM	0.14			0.17			0.21
				Dunnett p	0.999			0.794			0.593
				vs Cold-Si p	0.148			0.219			0.318
X-ray	#11	92.8	7.16	77.2	3.67	173.5	18.70	1.00	3.19	34.3	1.42
	#12	45.0	6.25	138.7	6.60	153.0	33.97	1.82	2.64	58.5	2.43
	#13	64.6	6.61	102.3	4.87	245.9	38.09	2.04	2.60	40.3	1.67
	#14	87.1	8.96	102.9	4.89	167.4	19.21	1.03	2.60	29.8	1.24
	#15	59.2	3.85	65.1	3.10	202.9	34.31	1.84	2.06	34.8	1.44
				Mean	4.63			1.55			1.64
				SEM	0.60			0.22			0.21
				Dunnett p	0.005			0.316			0.064
				vs Ctrl p	0.000			0.203			0.023
Day14		(fg)	(fg)	x1000/bact	fold change	(fg)	x10/bact	fold change	(fg)	x1000/bact	fold change
Control	#46	67.1	1.64	24.4	1.10	125.5	18.70	1.09	1.57	23.4	0.79
	#47	64.2	1.53	23.8	1.08	86.1	13.42	0.78	2.20	34.3	1.16
	#48	15.8	0.22	14.1	0.64	34.3	21.77	1.27	0.37	23.3	0.79
	#49	80.0	2.19	27.4	1.24	110.6	13.84	0.81	2.38	29.8	1.01
	#40	37.6	0.78	20.8	0.94	67.7	18.02	1.05	1.38	36.6	1.24
			Cnrl Avg=>	22.10	1.00	Cnrl Avg=>	17.15	1.00	Cnrl Avg=>	29.46	1.00
				SEM	0.10			0.09			0.09
Cold Si	#26	47.5	0.72	15.3	0.69	37.3	7.86	0.46	1.72	36.2	1.23
	#27	103.0	3.02	29.4	1.33	93.2	9.05	0.53	3.02	29.3	0.99
	#28	43.2	0.97	22.5	1.02	161.7	37.40	2.18	1.09	25.2	0.86
	#29	65.4	1.32	20.2	0.91	175.1	26.77	1.56	2.12	32.5	1.10
	#30	9.2	0.14	14.9	0.67	191.2	208		0.13	13.7	0.47
				Mean	0.93			1.18			0.93
				SEM	0.12			0.42			0.13
				Dunnett p	0.960			0.696			0.897
31Si	#36	87.8	4.63	52.7	2.38	94.1	10.72	0.63	1.96	22.3	0.76
	#37	58.9	1.13	19.2	0.87	141.5	24.04	1.40	1.34	22.7	0.77
	#38	48.9	1.37	28.0	1.27	63.1	12.89	0.75	1.62	33.2	1.13
	#39	106.9	4.77	44.6	2.02	90.1	8.43	0.49	3.04	28.5	0.97
	#40	82.1	1.88	22.9	1.04	81.9	9.97	0.58	2.29	27.9	0.95
				Mean	1.52			0.77			0.91
				SEM	0.29			0.16			0.07
				Dunnett p	0.186			0.993			0.942
				vs Cold-Si p	0.050			0.175			0.458
X-ray	#16	85.4	2.68	31.4	1.42	87.1	10.20	0.59	2.00	23.4	0.79
	#17	73.2	3.70	50.5	2.28	97.7	13.34	0.78	2.84	38.8	1.32
	#18	81.7	3.76	46.0	2.08	136.4	16.70	0.97	3.44	42.1	1.43
	#19	10.5	0.28	26.4	1.19	47.6	45.33	2.64	0.54	51.1	1.73
	#20	14.1	0.21	15.2	0.69	61.7	43.89	2.56	0.39	27.9	0.95
				Mean	1.53			1.51			1.24
				SEM	0.29			0.45			0.17
				Dunnett p	0.194			0.389			0.271
				vs Ctrl p	0.302			0.088			0.066

Lungs

Aqp1 (fg)	x1000/bact	fold change	Aqp5 (fg)	x1000/bact	fold change	Smad7 (fg)	x1000/bact	fold change
0.75	9.1	0.94	4.24	51.3	1.32	2.10	25.3	0.92
0.44	5.0	0.52	1.95	22.4	0.58	1.35	15.4	0.56
0.33	6.6	0.68	1.80	35.8	0.92	1.93	38.3	1.39
0.22	21.5	2.23	0.52	50.2	1.29	0.33	32.1	1.16
0.25	5.9	0.62	1.43	34.3	0.89	1.11	26.7	0.97
Cnrl Avg=>	9.61	1.00	Cnrl Avg=>	38.80	1.00	Cnrl Avg=>	27.57	1.00
		0.32			0.14			0.14
0.07	17.2	1.79	0.09	23.0	0.59	0.05	12.3	0.45
0.66	10.3	1.07	2.26	35.1	0.90	0.80	12.4	0.45
0.31	11.5	1.20	0.98	36.8	0.95	1.00	37.6	1.36
0.38	7.4	0.77	1.65	31.8	0.82	0.67	13.0	0.47
0.06	14.0	1.45	0.24	60.8	1.57	0.09	22.9	0.83
	Mean	1.26			0.97			0.71
	SEM	0.17			0.16			0.18
	Dunnett p	0.386			0.806			0.977
0.51	19.9	2.07	1.27	50.0	1.29	0.81	32.0	1.16
0.29	7.7	0.81	1.27	34.2	0.88	1.68	45.3	1.64
0.08	10.0	1.04	0.27	32.8	0.84	0.18	21.2	0.77
0.06	7.5	0.78	0.20	25.9	0.67	0.12	15.5	0.56
0.38	17.2	1.79	0.88	39.4	1.02	0.63	28.1	1.02
	Mean	1.30			0.94			1.03
	SEM	0.27			0.10			0.18
	Dunnett p	0.380			0.858			0.710
	vs Cold-Si p	0.451			0.446			0.126
0.66	7.1	0.74	5.48	59.1	1.52	1.86	20.0	0.73
0.46	10.1	1.05	1.57	34.9	0.90	0.97	21.6	0.78
0.61	9.5	0.99	2.01	31.2	0.80	0.94	14.5	0.53
0.62	7.2	0.74	1.83	21.0	0.54	0.91	10.5	0.38
0.54	9.1	0.95	1.88	31.8	0.82	0.68	11.5	0.42
	Mean	0.90			0.92			0.57
	SEM	0.06			0.16			0.08
	Dunnett p	0.773			0.868			0.998
	vs Ctrl p	0.377			0.356			0.013

(fg)	x1000/bact	fold change	(fg)	x1000/bact	fold change	(fg)	x1000/bact	fold change
0.67	9.9	1.10	3.36	50.1	1.01	1.86	27.7	0.98
0.53	8.3	0.91	2.76	42.9	0.87	2.57	40.1	1.42
0.12	7.8	0.86	0.49	30.9	0.62	0.22	14.0	0.50
0.65	8.2	0.90	5.93	74.2	1.50	1.92	24.0	0.85
0.42	11.2	1.23	1.86	49.6	1.00	1.32	35.0	1.24
Cnrl Avg=>	9.06	1.00	Cnrl Avg=>	49.54	1.00	Cnrl Avg=>	28.15	1.00
		0.07			0.14			0.16
0.52	10.8	1.20	2.86	60.2	1.21	2.09	44.0	1.56
0.68	6.6	0.73	3.86	37.5	0.76	2.29	22.2	0.79
0.25	5.9	0.65	0.94	21.7	0.44	0.59	13.7	0.49
0.38	5.8	0.64	2.50	38.2	0.77	0.95	14.5	0.52
0.05	5.3	0.59	0.24	25.5	0.52	0.04	4.1	0.15
	Mean	0.76			0.74			0.70
	SEM	0.11			0.14			0.24
	Dunnett p	0.996			0.974			0.964
0.86	9.8	1.09	5.16	58.8	1.19	2.68	30.5	1.08
0.36	6.2	0.68	2.78	47.3	0.95	1.25	21.2	0.75
0.39	8.0	0.88	2.36	48.3	0.97	1.72	35.1	1.25
0.93	8.7	0.96	4.77	44.6	0.90	3.71	34.7	1.23
0.90	11.0	1.22	5.62	68.5	1.38	2.56	31.2	1.11
	Mean	0.96			1.08			1.09
	SEM	0.09			0.09			0.09
	Dunnett p	0.891			0.552			0.578
	vs Cold-Si p	0.097			0.035			0.085
0.56	6.6	0.73	4.51	52.8	1.07	1.32	15.5	0.55
0.58	7.9	0.88	3.94	53.9	1.09	2.99	40.9	1.45
1.01	12.3	1.36	5.46	66.9	1.35	3.27	40.0	1.42
0.15	13.9	1.53	0.22	21.0	0.42	0.15	14.4	0.51
0.18	12.5	1.38	0.49	34.9	0.70	0.22	15.6	0.55
	Mean	1.17			0.93			0.90
	SEM	0.16			0.16			0.22
	Dunnett p	0.383			0.840			0.873
	vs Ctrl p	0.114			0.246			0.227
