# Supplementary material for: Gene Expression Changes in the Spleen, Lungs, and Liver of Wistar Rats Exposed to β-Emitted 31SiO2 Particles
Source: Int J Mol Sci. 2025 Mar 17;26(6):2693. doi: 10.3390/ijms26062693 (PMC11942150; doi:10.3390/ijms26062693)
Supplement: Supplementary file 1 [file ijms-26-02693-s001.zip › TableS2.pdf]

Table S2 - QRT-PCR data of the spleen of rats exposed to 31SiO2

Spleen

		bactin	Cdkn1a			Ccng1			Phlda3		
Day3		(fg)	(fg)	x1000/bactin	fold change	(fg)	x10/bactin	fold change	(fg)	x1000/bacti	fold change
Control	#41	102.9	0.95	9.20	1.10	80.0	7.77	1.03	2.71	26.34	1.10
	#42	105.5	0.67	6.31	0.75	57.7	5.47	0.72	2.16	20.49	0.86
	#43	198.4	1.84	9.29	1.11	193.5	9.75	1.29	4.20	21.16	0.89
	#44	100.3	0.79	7.88	0.94	83.1	8.29	1.09	2.68	26.74	1.12
	#45	63.3	0.59	9.25	1.10	41.9	6.63	0.87	1.56	24.61	1.03
			Cnrl Avg=>	8.39	1.00	Cnrl Avg=>	7.58	1.00	Cnrl Avg=>	23.87	1.00
				SEM	0.07			0.10			0.05
Cold Si	#21	137.2	1.45	10.55	1.26	156.1	11.38	1.50	2.84	20.69	0.87
	#22	114.0	0.80	7.04	0.84	119.2	10.46	1.38	2.34	20.57	0.86
	#23	44.6	0.28	6.33	0.75	22.1	4.96	0.65	1.00	22.47	0.94
	#24	61.7	0.53	8.62	1.03	47.8	7.75	1.02	1.50	24.39	1.02
	#25	88.5	0.83	9.41	1.12	101.8	11.50	1.52	2.28	25.78	1.08
				Mean	1.00			1.21			0.95
				SEM	0.09			0.17			0.04
				Dunnett p	0.826			0.350			0.947
31Si	#31	42.4	0.20	4.74	0.57	29.6	6.98	0.92	1.67	39.27	1.65
	#32	63.9	0.43	6.79	0.81	34.6	5.42	0.71	1.69	26.51	1.11
	#33	47.6	0.38	7.96	0.95	45.8	9.62	1.27	0.96	20.21	0.85
	#34	78.5	0.63	8.03	0.96	79.9	10.17	1.34	2.83	36.04	1.51
	#35	74.8	0.83	11.08	1.32	112.8	15.08	1.99	2.30	30.74	1.29
				Mean	0.92			1.25			1.28
				SEM	0.12			0.22			0.14
				Dunnett p	0.945			0.387			0.145
				vs Cold-Si p	0.308			0.455			0.030
X-ray	#11	90.5	2.90	32.05	3.82	120.8	13.35	1.76	3.53	38.99	1.63
	#12	90.4	2.57	28.46	3.39	121.2	13.41	1.77	3.37	37.28	1.56
	#13	59.6	1.81	30.38	3.62	73.0	12.25	1.62	2.53	42.49	1.78
	#14	103.4	3.91	37.78	4.50	183.8	17.78	2.34	4.97	48.08	2.01
	#15	117.8	3.04	25.81	3.08	194.7	16.52	2.18	4.93	41.83	1.75
				Mean	3.68			1.93			1.75
				SEM	0.24			0.14			0.08
				Dunnett p	0.001			0.001			0.000
				vs Ctrl p	0.000			0.000			0.000
Day14		(fg)	(fg)	x1000/bactin	fold change	(fg)	x10/bactin	fold change	(fg)	x1000/bacti	fold change
Control	#46	120.2	0.32	2.63	0.63	103.4	8.61	1.04	2.27	18.86	0.91
	#47	39.3	0.12	3.08	0.73	36.6	9.30	1.13	0.84	21.27	1.03
	#48	100.9	0.68	6.75	1.61	89.8	8.90	1.08	2.09	20.75	1.00
	#49	108.8	0.44	4.02	0.96	103.5	9.51	1.15	2.17	19.96	0.96
	#40	46.0	0.21	4.51	1.07	22.7	4.93	0.60	1.05	22.72	1.10
			Cnrl Avg=>	4.20	1.00	Cnrl Avg=>	8.25	1.00	Cnrl Avg=>	20.71	1.00
				SEM	0.17			0.10			0.03
Cold Si	#26	113.3	0.41	3.63	0.87	110.0	9.70	1.18	2.61	23.06	1.11
	#27	137.5	0.50	3.60	0.86	80.8	5.87	0.71	2.50	18.14	0.88
	#28	107.2	0.45	4.16	0.99	82.9	7.74	0.94	1.93	18.03	0.87
	#29	123.0	0.45	3.68	0.88	104.5	8.49	1.03	2.92	23.76	1.15
	#30	118.6	0.51	4.31	1.03	91.9	7.75	0.94	2.50	21.06	1.02
				Mean	0.92			0.96			1.00
				SEM	0.04			0.08			0.06
				Dunnett p	0.824			0.813			0.796
31Si	#36	133.7	0.42	3.17	0.76	107.9	8.07	0.98	2.87	21.46	1.04
	#37	118.8	0.46	3.85	0.92	86.0	7.24	0.88	1.90	16.03	0.77
	#38	69.3	0.24	3.43	0.82	33.0	4.77	0.58	1.00	14.45	0.70
	#39	124.3	0.88	7.12	1.70	80.2	6.45	0.78	2.14	17.24	0.83
	#40	82.0	0.32	3.89	0.93	48.2	5.88	0.71	1.87	22.82	1.10
				Mean	1.02			0.79			0.89
				SEM	0.17			0.07			0.08
				Dunnett p	0.655			0.985			0.990
				vs Cold-Si p	0.294			0.064			0.131
X-ray	#16	121.3	0.75	6.15	1.46	67.6	5.57	0.68	2.47	20.36	0.98
	#17	113.9	0.84	7.36	1.75	104.1	9.14	1.11	2.85	24.99	1.21
	#18	71.6	0.57	7.97	1.90	58.8	8.21	1.00	1.91	26.69	1.29
	#19	129.5	0.73	5.64	1.34	109.4	8.45	1.02	3.26	25.16	1.21
	#20	105.7	0.53	4.99	1.19	104.9	9.91	1.20	2.25	21.30	1.03
				Mean	1.53			1.00			1.14
				SEM	0.13			0.09			0.06
				Dunnett p	0.045			0.706			0.098
				vs Ctrl p	0.020			0.497			0.031
